# Fever

**DOI:** 10.1093/emph/eou014

**Published:** 2014-04-20

**Authors:** Elspeth V. Best, Mark D. Schwartz

**Affiliations:** ^1^Department of Anaesthesia, Royal Infirmary Edinburgh and ^2^Department of Population Health, NYU School of Medicine

## Fever

Fever, an elevation above normal body temperature, is a frequent symptom of many infections [1]. It results from the release of endogenous pyrogens such as prostaglandins and cytokines, which act on the anterior hypothalamus to increase the body’s temperature ‘set point’ [1]. Antipyretics like paracetamol (acetaminophen) appear to work by antagonizing prostaglandins, and are some of the most commonly used drugs in clinical practice [1].

Fever is usually regarded as a ‘noxious’ state that should be reversed in order to reduce morbidity, including convulsions and mortality [2]. This view is, surprisingly, not based on any experimental data [2] but rather a fear of any harmful effects [3]. Although antipyretics may ease discomfort during illness, this is likely due to the analgesic effects of most antipyretics rather than any reduction in temperature.

Few clinical trials provide evidence for the use of antipyretics. On the contrary, fever appears to be an evolved defence mechanism [2, 4], the suppression of which appears to have negative consequences [1, 5]. 

## Evolutionary perspectives

Fever is a highly regulated, primitive trait in most vertebrates and some invertebrates, with similar mechanisms suggesting it has been highly conserved [1]. Thus, fever likely has an important adaptive function in activating the immune system.

Increased body temperature leads to faster neutrophil migration, activation and proliferation of lymphocytes, production of cytokines including interferon and increased movement of lymphocytes [1]. There is also evidence that fever inhibits the growth of bacteria and replication of viruses by reducing plasma iron [6]. Fevers are also usually tightly controlled by a negative feedback loop [1] that prevents derangement and damage to the individual.

Studies have demonstrated that antipyrexials can have detrimental effects: increasing mortality when used in critical care and in influenza infections [7], increasing viral shedding [8] and slowing the rate of parasite clearance in malaria [9].

## Future implications

Current research is insufficient to warrant changing clinical practice but indicates the urgent need for further studies. 

With a greater understanding of evolved defence mechanisms, clinicians will be able to better comprehend when these responses can be altered and when they should be preserved and help demarcate the instances in which the febrile response is truly dangerous and should be treated promptly [10].
